# Are Front-of-Pack Labels a Health Policy Tool?

**DOI:** 10.3390/nu14040771

**Published:** 2022-02-11

**Authors:** Luca Muzzioli, Claudia Penzavecchia, Lorenzo Maria Donini, Alessandro Pinto

**Affiliations:** Department of Experimental Medicine, Sapienza University, 00185 Rome, Italy; muzzioli.680093@studenti.uniroma1.it (L.M.); penzavecchia.1048244@studenti.uniroma1.it (C.P.); lorenzomaria.donini@uniroma1.it (L.M.D.)

**Keywords:** front of pack labeling, nutrition labeling, nutrient profiling, FOPL, non-communicable diseases, traditional foods, food industry

## Abstract

To stem the increasing incidence of non-communicable diseases (NCDs) and obesity, front-of-pack labels (FOPLs) have been developed since 1989. Whereas several countries have already adopted one voluntarily, the European Community wants to harmonize an FOPL system that will be mandatory for all member states. The purpose of this narrative review is to describe what could be achieved or not by FOPLs, and to discuss if there is enough evidence to establish whether such labels are effective in modifying purchasing behavior, in directing individual dietary patterns towards a healthy and sustainable diet, and in reformulating food products by the food industry. Non-directive FOPLs, which are still under study, appear to be informative and well-accepted by consumers even if they require a cognitive effort. Conversely, directive FOPLs are supported by several studies, but they are mostly conducted in simulated scenarios and/or performed as retrospective studies. Nevertheless, directive FOPLs are rated as an intuitive tool, and they have demonstrated a high capacity to help consumers rank food products as more or less healthy. In conclusion, directive and non-directive FOPLs convey different messages. No FOPL individually can be considered exhaustive in relation to all the objectives outlined in this narrative review, and therefore, the development of a model synthesizing both messages is advisable. Many questions remain open, such as the possibility of reformulating pre-packaged products, how to deal with traditional products, and the impact on the incidence of NCDs and obesity. In the light of the complexity of factors that condition consumption choices and health, none of the current FOPLs can be considered a health policy tool on its own. The possibility of development remains open, but as the state of the art, these tools do not seem to be able to achieve all the European Community goals together. We can speculate that they could meet these goals only if they are integrated into a multi-tiered, structured health policy intervention.

## 1. Introduction

Packaging is the first contact of a consumer with a product, and the characteristics of packaging often determine the interest in the product itself. Traditionally, packaging is perceived as a means of protection for the product during the process of distribution, transport and storage; currently, it is increasingly becoming an effective way to communicate with the consumers to answer an increasing demand for information about the relationship between diet and health. This information could help consumers make the right choices when buying food products. An analysis of packaging features showed that the most important information sought by consumers has to do with shelf-life, price, and product composition, but they are also interested in information on caloric content, individual nutrients, and the food product’s origin [1].

In this general context, since 1989 the number of public and private initiatives to create front-of-pack labels (FOPLs) for packaged products has increased, not only for matters of food safety but also due to the growing evidence on food-related chronic degenerative diseases, including the global obesity epidemic [2]. Although it is recognized that non-communicable diseases (NCDs) and obesity have a multifactorial etiopathogenesis, and it is unlikely that a single prevention strategy will significantly change the risk of becoming ill, providing information and guidance to consumers could be a first tool to move towards healthier food choices. In this perspective, the back-of-pack label (BOPL) information is generally considered by consumers to be “inaccessible and difficult to understand” [3], and therefore, FOPLs have started to be developed. The FOPL strategy, after being used independently by several countries around the world, was proposed by the European Commission at the EU level to address the problems linked to an unbalanced diet and the health needs of the population [2]. Alongside this, other strategies have been proposed and partially implemented in Europe: “reformulation agreements, marketing restrictions on foods high in fat, salt and sugar, public procurement for healthy food products, taxation of sugary drinks” [2].

Since 2004, WHO has proposed FOP labeling “as a tool for promoting healthy diets and preventing obesity and diet-related NCDs”, providing recommendations for FOPL models to be adopted, and suggesting that they should be supported by a communication strategy and monitoring actions [4,5,6,7]. In 2019 the Codex Alimentarius also provided some guidelines for the formulation and placement of FOPLs on packaging, as well as general principles of comprehensibility and harmonization with other nutritional information and communication standards. In this perspective, the FOPL system must be adequately tested and validated, and, once adopted, it must lead over time to a real and significant improvement of health status in the European Community [8].

FOPLs, therefore, have two main objectives: (1) to communicate complex information to consumers by simplifying it into a standardized format to guide, inform and/or steer consumer food choice and behavior; (2) to stimulate the food industry to reformulate some products in a healthier direction. Over time, the goals will extend to the possibility of convincing or guiding people to change their purchasing choices by including a health assessment of the food product. Although not mentioned, it is clear that one aim of FOPLs is also to help consumers make informed choices in terms of overall dietary patterns that can contribute to people’s health. For this reason, most studies on FOPLs assessed the potential health effect using simulations and mathematical models.

Compared to the nutrient declaration (usually placed on the back or side of the package, BOPLs), FOPLs can vary in design (colors, shape, size), in the message they convey, in their public health objective (prohibitive, prescriptive, or both), and in their focus on specific nutrients (i.e., nutrients considered “critical” to health, such as sodium, saturated fatty acids (SFAs), trans fatty acids (TFAs) and sugars). Some FOPLs provide the percentage of energy and nutrients in relation to the standard requirement of 2000 kcal/day, while others provide ratings of the content (low, medium, high) of specific nutrients. Others calculate a score indicating the global quality of a product. Some are based on a standard value of 100 g or 100 mL, and others refer to the portion available for consumption.

The purpose of this narrative review of the literature is to clarify the goals and the objectives that could and could not be achieved by FOPLs, and thus to evaluate whether there is significant evidence to establish whether such a nutritionally informative model can be considered effective: (1) in modifying purchasing behavior, (2) in guiding individual dietary patterns towards a healthy and sustainable diet, and (3) in reformulating food products by the food industry. 

Research was conducted by searching PubMed articles published after 2004, excluding non peer-reviewed papers using keywords such as “front of pack labeling”, “front of pack health”, “nutrient profile”, “front of pack impact”, “(front of pack) AND (eating pattern)”, and ”traditional food product labeling”. From the global results obtained, we excluded articles not relevant to the three main purposes of our review. Furthermore, we limited our research at first to systematic reviews and meta-analyses, and then we broadened the search to narrative reviews as well as observational and experimental studies. We also sought documents and opinions of the major agencies and organizations that provide advice and support to EU policy makers (WHO, EFSA, JRC, FAO, Codex Alimentarius) as well as EU regulations and reports.

## 2. The Background of FOPL Development

The Codex Alimentarius Commission has identified three types of information to be included in nutrition labeling [8]: nutrient declarations, health and nutrition claims, and supplementary nutrition information. Nutrient declarations are mandatory and standardized lists of the amounts of nutrients contained in food products or beverages and are usually placed on the back or on the side of the package (BOPLs). All of the ingredients in the food, in decreasing order of weight, must be displayed on the label, including information about the energy value and the amount of fat, SFAs, carbohydrates, sugars, protein and salt, in a tabular or linear format. The mandatory nutrient declaration should refer to 100 g or 100 mL amounts and, in addition, portion-based declarations are allowed. The package side usually chosen is the back, even if not specifically requested by Regulation (EC) 1924/2006, which only states that nutrition information shall be included in the same field of vision. This information is not intended to provide quantitative knowledge on what should be eaten to be healthy. Nutrition and health claims suggest or imply that some food “has particular nutritional properties including, but not limited, to the energy value and to the content of protein, fat and carbohydrates, as well as the content of vitamins and minerals” or other health promoting components [8]. Supplementary nutrition information is intended to help consumers to understand the nutritional value of the food and the meaning of the nutrient declaration. According to the Codex Alimentarius, all of this information should be accompanied by a consumer education program. However, no label should deliberately imply that a food has a nutritional advantage over a food that is not labeled.

According to Regulation (EC) 1924/2006, in order to be able to provide nutrition or health claims, the food has to meet the nutrient profile (NP) criteria.

The NP (Regulation (EC) 1924/2006 Article 11) is a tool for identifying the intake thresholds of specific nutrients (such as fat, SFAs, TFAs, salt/sodium and sugars—whose excessive intake is not recommended—as well as poly- and monounsaturated fats, available carbohydrates, vitamins, minerals, proteins and fibers, above which no health claims are allowed, in order to prevent consumers from considering “healthy” some foods that may adversely affect a nutritional dietary pattern [9]. 

According to WHO, “FOPL systems must be underpinned by a specific nutrient profiling model” [10]. Therefore, NP was used as a basis for the development of some FOP labeling models (e.g., Multiple Traffic Light, Health Star Rating, Nutri-Score), resulting in the attribution of specific symbols, colors, or nutritional scores. They can be useful for regulating the marketing of products to specific population groups (e.g., children), preventing NCDs, and promoting consumer health [11].

In support of a decision the EC was scheduled to make in 2009, EFSA (2008) summarized the key points of its scientific position in an opinion report on nutrient profiles [9].

The choice of food components to be included in the NPs should be established based on their impact on the public health of EU citizens. These components were identified by the WHO in 2003 and included in the dietary guidelines of several EU countries as positively (energy, SFAs and TFAs, sodium, simple sugars) or negatively (dietary fiber, fruit, vegetables, potassium, omega-3 polyunsaturated fatty acids, vitamin D, calcium) related to the risk of chronic NCDs and obesity. In most EU countries, intake of these food components is misaligned with dietary guidelines. However, overly complex nutrient profiles should be avoided, and the total number of nutrients displayed or included in the NP should be limited. EFSA’s opinion suggests that a single shared NP model should be applied to all foods, with possible limited exemptions for those that, for example, play a key role in national dietary habits and traditions. Several NP models have been developed (e.g., Ofcom/FSA NP model, WHO-Euro model and Health Canada Surveillance Tool system), but there is still no consensus on which of these may be assumed as gold standard to objectively define the healthiness of foods [12].

There are some inherent difficulties in establishing nutrient profiles at the EU level, such as the application of nutrient intake recommendations for the general diet to individual foods, the lack of uniform data on the composition and consumption of foods across the EU, and differences in nutrient intake recommendations and dietary guidelines within EU countries [9]. 

## 3. FOP Labeling in Europe

Regulation (EC) 1924/2006 (on nutrition and health claims made on foods) and Regulation (EU) 1169/2011 (the Food Information to Consumers or “FIC”) have sought to harmonize the food labeling system in the EU. According to the EC regulations, food labels should not be false or ambiguous, should not give rise to doubt about safety and/or nutritional adequacy of foods, should not encourage or condone excess consumption of a certain food or refer to changes in bodily functions, and should enable consumers to make an informed choice [2,9]. 

The “FIC” regulation is meant to protect consumers against misleading advertising and to defend producers against unfair competition. In addition to regulating the mandatory nutrient declaration, it sets out rules for the voluntary information that usually concerns the most relevant ingredients listed in the nutrition declaration, in order to help consumers see and understand this information more easily. A FOP labeling model shared on a Europe-wide level could be applied only after verifying compliance with eating habits and gastronomic traditions for all European countries, since it plays a significant role in determining the overall nutritional pattern. 

The EU’s Joint Research Centre (JRC) [3] states that currently, six FOP models have been developed or approved by the public sector. Other FOPL models are in use in the private sector. 

FOPLs can be distinguished according to the complexity of the information provided (displaying nutrient-specific information or a global judgment on the whole product) and their “directionality”, i.e., the kind of guidance or evaluative message with regard to healthiness. On these bases, they could be categorized as follows:

Non-directive labels that provide information such as the name of the nutrient, the amount in grams, and the percentage of the total (e.g., Reference Intakes, Nutrinform Battery)

Semi-directive labels that not only provide nutritional information but are completed by an evaluative element such as a color, a word, or a sign that gives additional information on the healthiness level of single nutrients, emphasizing them (e.g., the English traffic light or Multiple Traffic Light—MTL, Warning Signs which may feature the octagon “stop” or the words “rich in”)

Directive labels, that include little information, often aggregated in a single symbol (e.g., Swedish Keyhole, Nutri-Score) and combining several criteria. They give information about the healthiness of the product, expressing judgments, opinions and/or recommendations, without providing specific information on single nutrients.

Warning signs are not strictly considered FOP labels, but they are nevertheless used in studies when testing different FOPLs. In the same way, nutrition and health claims are not technically FOPLs, although they may, in part and in some circumstances, be assimilated into, or used in conjunction with, other FOPLs. Table 1 summarizes some of the main FOPLs available in the EU and worldwide (modified from Delhomme, V., 2021 [13]). 

In Table 1, non-directive labels are classified as reductive, while semi-directive and directive labels are included in the evaluative category.

The magnitude of the introduction of FOPLs in the EU market is neither clear nor up to date. The JRC [3] reports an estimate from 2008–2009, when about 48% of products (chosen from five categories) displayed an FOP label (the percentages seem to be lower for endorsement/health logos, since they are limited to the healthier options within a food category and are unlikely to be displayed on all products).

It is essential to underline that:

the European Commission plans to adopt a harmonized FOP labeling system that will be mandatory for all member states by 2022, although this system should be based on an NP that is still awaiting validation and definition; 

with regard to the nutrients relevant to public health, according to EFSA’s opinion, it is worth highlighting that different labels currently take into account different compounds, including nutrients that are not directly recommended by national dietary guidelines or by WHO;

with regard to the reference quantity, most of the evaluative/directive FOPLs refer to 100 g or 100 mL, which is not useful for directing the consumer towards a choice that is congruent with personal needs.

## 4. Directive FOPLs and Nutrient Profiling 

Whereas non-directive FOPLs rely directly on nutrition facts, directive labels are based on nutrient profiling systems. Nutrient profiling has been called “the science of classifying or ranking foods according to their nutritional composition for reasons related to preventing disease and promoting health” [5]. WHO considered NPs a useful tool for a variety of applications and to be a critical tool for the implementation of restrictions on the marketing of foods to children [14]. Within the European area, five nutrient profiling systems have been developed: by the United Kingdom (the FSA-NPS), Denmark, Norway, the WHO Regional Office for Europe (the WHO-Euro model), and the EU Pledge Nutrition Working Group. To date, no gold standard has been assessed even if comparisons have been made [15,16]

Nevertheless, the FSA-NPS, which was developed in 2005 by the British Food Standards Agency, is the most studied. It was implemented to establish restriction policies for television advertisements about foods and beverages aimed at children, i.e., to restrict the broadcasting of advertisements of products high in fat, SFAs, salt and/or sugar [17,18,19,20]. The algorithm generates a score for foods and beverages, computing the nutrient content for 100 g of energy, total sugar, SFAs, and sodium, from which it subtracts the content of fruits, vegetables, nuts, fibers, and protein. Eventually, the resulting score relies on a discrete continuous scale ranging from −15 (healthiest) to +40 (least healthy). The FSA-NPS algorithm is currently the basis for two different FOPLs: the Nutri-Score, which uses a 5-color scheme and letters from A (best score) to E (worst score), and the Health Star Rating, which classifies foods from 0.5 (worst score) to 5 stars (best score) [18,19,20].

On the other hand, the WHO-Euro model was also created in the first decade of the 2000s to regulate products suitable or not eligible for advertising to children. It has to be mentioned that it was developed on the Danish and Norwegian systems instead of the FSA-NPS due to the fact that the former are based on food categories rather than a scoring system. “Category-specific models are considered easier to adapt or modify than models based on scoring, which is an important consideration for a regional model that countries will be looking to use nationally” [21].

## 5. The Impact of FOPLs on Food Choice and Consumer Behavior

The impact of FOPLs on purchasing behavior and accuracy of food classification can be analyzed in terms of consumer attention, acceptance and understanding. 

Attention could be defined as “the degree to which consumers focus on stimuli within their range of exposure” [22]. It is usually measured with eye-tracking tests and can be gained by the use of advertisements and dynamic packages [22]. Acceptance is usually determined or affected by the quality/price ratio, popularity, taste, familiarity, ideas about food quality and safety, and label understanding [22,23]. Acceptance can be influenced by external factors such as preexisting attitudes, ideas and knowledge towards and about foods [23,24]. The understanding of a label at the same time can influence consumer attention, acceptance and trust in the product, and it can be mined by technical terms [23].

FOPLs, as well as other types of labels and claims, are not always correctly understood by the public. Sometimes the problem lies in poor consumer nutrition literacy; at other times the influence that traditional and social media exerts on consumers and the establishment of new food trends can lead to misconceptions, lifestyle “musts” and a general confusion about the labels. For this reason, “healthiness” can have different meanings to different people, and differently drive them towards purchase. For some people, it is related to the amount of sugar, energy, or salt in a product, whereas for others, it may refer to the presence or absence of gluten, to whether a product is labeled as organic or not, if labeled as “free from” for a specific component/nutrient or, conversely, with “100%” content [25,26,27,28,29,30,31]. An example of this can be observed by considering how the same concept changed within the same group over time (e.g., how our attitude towards fats and sugars has changed over the last 50–60 years [32,33]).

In an attempt to better understand the level of comprehension among consumers and the impact of these labels, experimental studies, sales data and shopping cart monitoring have been used to assess the impact of FOPLs on purchase intentions or actual purchases, in controlled or real-life contexts. The findings are supported by the replacement of foods displaying an unfavorable FOPL with those displaying a more favorable one. Color-coded FOPLs, with or without a score, seem to facilitate better food choices and improve the nutritional quality of the shopping basket.

Some studies and a few literature reviews (summarized in Table S1, see Supplementary materials) show a high discriminatory capacity of the Nutri-Score for most food groups. In the simulation studies, fruits and vegetables were usually correctly classified by the participants in the “healthiest” categories of the Nutri-Score (“A” and “B”), while products rich in sugars and animal fats were classified in the “less healthy” categories (“D” and “E”) [34]. However, the ability of the Nutri-Score to discriminate the nutritional quality of foods and beverages between and within food groups was found to be inconsistent with the dietary guidelines in some countries (Spain and the Netherlands), especially with regard to specific food groups (cheese, beverages, ready meals, sauces, soups and seasonings), and, therefore, these countries asked to adjust the scoring system [35,36]. This underlines the need for considering the role and nutritional impact of the different food categories in each country, due to local eating habits and traditions. Moreover, there is a gap between the assumption that FOPLs could help consumers rank foods on a health benefit scale, and the assertion that consumers make better food choices, thus reducing their overall risk of NCDs or becoming obese [37].

Hagmann and Siegrist (2020), in an experimental study, compared the MTL (a version adapted for the German market), the Nutri-Score and the Nutrition Facts tables (per 100 g) of 15 snacks currently purchased in Switzerland. The selected FOPLs compared to no label seem to help consumers in identifying healthier snacks. Most of the participants, at the end of the simulation, declared themselves in favor of MTL in Switzerland and considered the Nutri-Score the least useful among the tested models. This result is probably due to the greater familiarity with the MTL model among the consumers recruited in the study, as well as to the broader nutritional information, in comparison with the more synthetic approach that characterizes the Nutri-Score [12].

It must be noted that several experimental trials assumed that all food products available for purchase have a label displayed; nevertheless, FOPL application is currently not mandatory, and as a result, product comparisons by the consumer are not always possible. Even in countries where FOPLs are now in use, this evaluation is often obtained using mobile applications (such as the Open Food Facts crowdsourcing database for Nutri-Score) that do not allow one to analyze the representativeness of the food sample, either in terms of the number of products or market share, given the difficulty of knowing if what these databases show corresponds to what consumers usually buy [17].

Some studies do not show statistically significant differences in purchasing behavior and in the accuracy of food classification in the presence or absence of FOPLs, especially when the label is not supported by an adequate information program [38]. It is also necessary to underline the lack of uniformity among the study designs: nutritional values and product names and/or brands are not always included, making it difficult to compare study results and to state that one FOPL is more effective than another. These results do not represent a realistic scenario that would allow us to assess the level of understanding of the products’ quality among consumers, because they do not correspond to a real-life behavior [39]. Biases and factors influencing food purchases and the determinants of consumption profiles influencing food choices also need to be considered.

Some studies seem to point out that FOPLs lead the consumer to ignore the nutritional declaration usually found on the back of the package (BOPL) [40,41,42]. Whether this is a success or a failure from the perspective of the population, food education has to be judged. Furthermore, virtual simulation studies usually evaluate the ability of consumers to choose the “best” products within food categories considered to be “less healthy” (i.e., snacks, pizza, breakfast cereals, cakes, biscuits). This can make us assume that the ability to rank is limited to these food categories, and that the impact on purchases is not the same for all categories. However, in real life, products evaluated as “negative” are often chosen regardless of the awareness of their quality, as they are considered “hedonistic” and subjected to emotional and irrational decision-making processes [43,44,45,46,47,48]. Studies highlight that the “healthiness” of a food is usually not a relevant criterion in the choice of purchasing snacks. For these foods, parameters such as palatability, price, convenience and brand seem to be more important [49,50,51,52]. This would explain why the Nutri-Score appears to not have a significant impact on snack choices [12]. This is highlighted when FOPL understandability is not reflected in consumer choice, as in Fialon et al. [39] and Vandevijvere S. [53]: they studied respectively a sample of Italian and Belgian subjects, and the Nutri-Score was found to be the best in helping consumer to categorize foods, but it was not associated with a shift in food choices between food categories. The same result was found in Hamlin et al. (2020), where the HSR label was not associated with a change in breakfast cereal choice among adolescents [54].

Therefore, the hypothesized scenario appears to be more ambitious than what current evidence suggests to be a realistic impact of FOPLs: these simulations have to be seen as an idea of what dietary improvements could be achieved under ideal circumstances [3], i.e., in the “best of all possible worlds”. In fact, evidence of the impact on purchasing behavior is limited, and it suggests a small beneficial effect on “on-site” purchases. Real-life buying decisions are influenced by a multitude of factors (other than FOP labels) that make it difficult to isolate the specific effect of FOPLs.

## 6. The Impact of FOPLs on Dietary Pattern

Even if not clearly specified in their objectives, some FOPLs aspire to modify the consumer food choice, determining a modification of the individual dietary pattern towards a healthier diet [3]. This means that their aim is not only to give information about food, but to perform an intervention of primary prevention. Therefore, one aspect of the effectiveness of FOPLs is their ability to help consumers make healthier food choices at the point of purchase (PoP) and consequently reduce the hazard ratio (HR) of NCDs. 

To investigate this hypothesized connection for directive labels based on the FSA-NPS, a dietary index (DI) was developed. It aggregates food (or beverage) scores at the individual level with the following equation:

$$FSAm-NPS\;DI=\frac{\sum_{i=1}^{n}\left(FS_{i}E_{i}\right)}{\sum_{i=1}^{n}E_{i}}.$$

where FS_i_ represents the food (or beverage) score, and E_i_ represents the energy intake from the food or beverage. Higher index values indicate poorer nutritional quality [55].

Associations between dietary index (DI) and the risk of cardiovascular disease (CVD) [56,57], cancer [58,59], overweight and obesity [60] were studied in volunteer subjects in five prospective cohort studies. The results suggested that lower dietary index (DI) scores are associated with a lower risk of CVD, cancer and weight gain (in men), showing that the FOPL model based on the FSA-NPS could help consumers shift their diets towards a lower risk of becoming sick [3]. However, the FSA-NPS does not consider food portion size, frequency of consumption and variety, all factors that, in correct combination, define a dietary pattern [61]. Thus, relating a dietary index to an algorithm (that is not a dietary model) could lead to unexpected results such as high-protein diets with low FSA-NPS-DI scores (which correspond to high dietary quality). However, it is well known that excessive red meat intake is positively associated with all-cause mortality [62,63,64,65] and that high consumption of food from animal sources is related to the increase in global warming [66,67] due to greenhouse gases such as livestock-produced methane [68]. Nowadays it is also mandatory to discuss healthy diets from a sustainability-related point of view.

Currently there is no clear and irrefutable evidence that any FOPL can lead to significant changes in dietary patterns towards prevention of food-related NCDs, also because it is extremely difficult to carry out studies that demonstrate a clear causality relation. A person’s health cannot be determined exclusively by the diet, and the adjective “healthy” cannot apply only to the diet: it would be more accurate to say that a person or a group of people are “healthy” if they lead a healthy life [11,69]. Nevertheless, the use of nutrition facts and compliance with a healthy diet such as the Mediterranean diet [70,71] seem to be positively correlated.

From modeling studies, it is possible to infer that habitual intake of products with more favorable nutritional profiles (i.e., better scores and more positive FOPLs) probably reduces the intake of energy and nutrients that have to be restricted, while increasing the intake of beneficial and recommended nutrients. 

Non-directive FOPLs provide consumers with information about the contribution of food to the energy and nutrient intake within a standard diet of 2000 Kcal/day. Among other labels, the NutrInform Battery was developed in Italy in 2018–2019 with the aim to help consumers make informed decisions about their daily food consumption, and it consists of a battery that fills proportionally to the nutrient composition of the foods. As the NutrInform Battery is a recent proposal, there are still limited data on its informative power and likability. Mazzù et al. [72,73,74] started testing the Battery in Italy and in seven EU countries, comparing it with the Nutri-Score. From early data, it seems that the Battery is perceived as more informative and useful than the Nutri-Score in understanding food composition. In Europe, a study of 2776 people using mock products (on which no brand name appeared to avoid influencing the subjects with parameters such as brand loyalty) in four categories (sauces, yoghurt, biscuits and crackers) tested both FOPLs in terms of the comprehensibility of their design, their ability to help consumers make purchasing decisions, and their complexity and pleasantness. The results show that the Battery seems better at informing, is more credible and easier to understand, outperforming the Nutri-Score in subjective understanding, with some non-significant variability between countries in terms of likability. The Battery symbol has been criticized for being counter-intuitive and requiring an interpretive effort [75]. This statement, however, requires more studies on larger populations and possibly in a real-life setting; otherwise, it would remain hypothetical, based on the mere interpretation by those who find the symbol misleading on a subjective level.

Such non-directive labels, as well as reference intakes, require more cognitive effort from the consumer, but in the long run, this could favor an increase in nutrition knowledge and more balanced dietary patterns. Of course, none of this will be possible unless we invest in serious and structured nutrition education campaigns run by professionals throughout the EU community.

On the other hand, directive FOPLs can yield different results depending on their approach. Warning labels (WLs) are based on a negative stimulus, and they give information about food enriched in a specific nutrient. In some cases, such as for sugars, due to a dose–response relation, it could be difficult to find a threshold for the negative effect on health [76]. MTLs provide information on nutrient composition per serving, similarly to the NutrInform Battery, adding green, yellow, or red color to each nutrient labeled for comparison to dietary reference values (DRVs). Issues regarding thresholds in use are the same as those mentioned for WLs. Lastly, directive FOPLs such as Nutri-Score and HSR give a simplified output: colors and letters for the Nutri-Score, and a starred score for HSR. These labels appear more appealing and easier to understand at PoP [77,78,79].

However, an aspect of concern comes from the across-the-board approach of the FSA-NPS-derived FOPLs. Comparing all foods on a unique scale may be confusing and alter individual dietary patterns at PoP in an unknown and not always healthy direction, especially if the FOPL does not give any information about portion and frequency of consumption. For example, a possible scenario is that a food rich in salt could be exchanged with a food rich in protein only because the latter has a better score. In particular, the Nutri-Score label, which does not show any amount of any nutrient on the label, could force consumers to use the BOPL if in need of a tailored diet (e.g., a reduced salt diet for hypertensive issues or reduced sugars for hyperglycemic issues). Furthermore, Nutri-Score divides all foods into five huge categories, which may not give consumers enough information to find a healthier alternative within the same food category due to the fact that less healthy categories cover a range of eight or more points (e.g., chocolates are all ranked in the E category). When at PoP, consumers could completely avoid buying some foods or some categories, reducing their food variability, or conversely, when they decide to consume a less healthy product with a hedonistic motivation, this could lead to intake ad libitum due to the lack of an indicated portion size. On the other hand, if scores were normalized for each food category, consumers could misunderstand the ranking system on relative values and consume some products more than necessary. 

In this scenario, FOPLs that show serving size and portions and/or relative nutrient amounts in relation to DRVs, such as MTLs and the NutrInform Battery, can give the consumers an instrument to include all foods in a healthy dietary pattern. These FOPLs allow people not only to make an informed choice at the time of purchase, but also at the time of consumption at home, depending on what they have already consumed during the day or what they expect to consume; it helps them to choose and consume foods considering their overall diet, and to develop a daily diet by “balancing” the food products they choose. Information given by this type of label is factual and concrete, and not revised by the creator of the symbol [80]. Consumers, once they have bought their groceries, would be able to combine products according to what they have in their pantry and what they have already eaten during the day. Conversely, directive FOPLs do not give consumers this opportunity.

With regard to the assumed health effects of the FOPLs, there is not yet enough evidence to draw conclusions as to whether they help lower the risk of developing NCDs [2,6].

Indeed, the meta-analysis by Ikonen et al. (2020) [6] seems to suggest that FOPLs succeed in meeting the first part of the set objectives, i.e., increasing the proportion of consumers who can notice and understand product information, but it is not clear whether they are useful in helping consumers to make healthier choices; the ability to classify products found on the market does not automatically translate into their ability to make choices to achieve healthy patterns and conscious eating habits [81].

A Spanish study found that a higher FSA-NPS-DI score (corresponding to poorer quality of the foods chosen, with higher consumption of sweets, processed meats, fast food and sugary drinks and lower consumption of vegetables, fish and poultry) was associated with a higher mortality rate from all causes and cancer (but not from cardiovascular diseases) [82]. Moreover, in this study, the classification of foods according to the Nutri-Score was consistent with adherence to the MedDiet score (Pearson’s correlation coefficient, r = −0.45) and the Spanish guidelines (r = −0.51).

Ikonen et al. [6] point out how the effects of FOPLs have changed over time and how, compared to the first studies carried out, the most recent ones show weaker effects on food choices and behavior. Furthermore, differences between published and unpublished studies seem to have been found, “with unpublished studies showing more negative effects on healthy choices (*β*  =  −0.236, *p*  < 0 .001) and consumption (*β*  =  −0.238, *p*  < 0 .001), when controlling for other factors” [6].

A macro-simulation study that used the Preventable Risk Integrated Model (PRIME), estimated the impact of FOPLs on deaths from NCDs, suggesting that they could delay or prevent 3.4% of cases on average, with some differences between labels, and the greater results were achieved by Nutri-Score and HSR [83]. However, in different settings, HSR has been criticized for not being particularly useful in influencing consumer choice, especially in purchasing sugar-rich products [54].

As the JRC points out [3], many of the studies analyze a consumer sample in a steady state model instead of a dynamic one, and for this reason the consumer is depicted as unable to modify his/her eating habits and lifestyle. These studies examine FOPLs in an isolated condition, unaffected by external factors, leading to a potential overestimation of the benefits by overlooking confounding factors such as compensatory consumption, increased physical activity or consumption of foods perceived as more nutritious or healthy. In addition, the lack of data on nutrient intakes has often required the use of outdated consumer surveys, raising questions about the representativeness of the present population behavior [3]. 

## 7. Impact on Food Industry

Regarding the impact of FOPLs on the food industry, theoretically they could and should lead to improvements in product formulation and nutritional profile. One potential risk is that reformulation occurs only for nutrients that are included in the FOPL algorithm—nutrients that paradoxically could be replaced by others not necessarily healthier but that are not included in the algorithm. For example, if SFAs are eliminated and replaced with carbohydrates, the health impact would likely be neutral, at best [84].

With respect to directive FOPLs referring to 100 g or 100 mL, two different products classified as “red” (unhealthy) can be considered equal by the consumer despite having highly different amounts of unfavorable nutrients [85]. In fact, directive summary labels such as the Nutri-Score show a global assessment that is not differentiated by nutrient, hiding information about the content. The following scenario could occur: by adding several products to the shopping cart, even if all “green”, the recommended limit on intake of an unhealthy element, for example salt, can be exceeded.

Some industries have already been moving along this path, calculating how broad product reformulation should be [86,87]. In these studies, a different NP was used, namely the so-called Nestlé Nutritional Profiling System (NNPS). The NNPS, in contrast to the FSA-NPS, is category-specific, and it calculates nutrients’ targets per serving instead adopting an across-the-board and per 100 mg/mL approach. This means that the former ranks foods only within their categories, while the latter compares individual foods with each other. Despite this difference, NNPS showed that the most common nutrients in need of reformulation were SFAs and total fats and, in compliance with NNPS standards, that SFAs, sodium and added sugars should be reduced in content (by 10%, 8%, and 6%, respectively). 

At least two aspects require further discussions: on one hand, whether packaged product reformulation would be possible or not, it does not interfere with the usefulness of directive FOPLs, and negative outcomes of this secondary purpose sill leave intact the reliability of directive FOPLs in achieving their primary aim. On the other hand, what appears clear is that product reformulation needs the combined effort of scientific research and industries working together to drive packaged products towards a healthier composition.

In this context, it has to be mentioned that there are products such as traditional food products (TFPs) that cannot be reformulated for two reasons: on the one hand, bromatological reformulation is likely to alter organoleptic features, while on the other hand, TFPs are regulated by Regulation (EC) 510/2006, which defines TFP standards, gathering them under collective trademarks (e.g., PDO, PGI, TSG). Council Regulation (EC) 509/2006 gave the following definition of “traditional” in relation to foods: ‘‘Traditional means proven usage in the community market for a time period showing transmission between generations; this time period should be the one generally ascribed as one human generation, at least 25 years’’. Hence, TFPs differentiate from industrial foods because they must be linked to the gastronomic traditions of a specific territory and are persistent over time [88].

Europe cannot be regarded as a homogeneous food culture, because noticeable differences exist not only at a national level but also at a more regional/local level in terms of food preferences, habits, food-related behavior, and attitudes [89]. Compared to Scandinavian and Benelux countries, Southern European countries such as Italy, Spain and France have a higher number of collective quality marks that can be regarded as possible candidates for registration as a PDO or PGI [90]. These products are part of regional and traditional food culture from more than three generations, and they are key factors to protect and preserve in an economic-productive scenario of the Mediterranean basin [91], even though, on the nutritional perspective, TFPs often have a high energetic density. Because of policy and market interests, traditional foods have become increasingly attractive from an industry perspective, especially for small and medium-sized enterprises. Emphasizing product attributes generated by regional characteristics of the manufacturing area or by the use of traditional production practices, creates new opportunities for marketers [92]. Moreover, cultivation of local raw materials and ingredients, which are mostly used in the production of traditional foods, contributes to the development of a more sustainable environment, protecting rural areas from depopulation and providing a wider variety of food choices for consumers [93]. 

Since TFPs are often energy-dense foods, a classification with a healthy scale on a 100 g basis instead of a portion basis could lead to an excessive fall in their consumption, with a subsequent loss in food variability and regional traditions and a breakdown in local economies and employment in rural and fishery areas. Furthermore, the exclusion of some foods (such as TFPs) from the FOP labeling system could create disparities between products available on the food market, invalidating the primary objective of this health policy instrument with misleading information at PoP, where consumers could be expected to compare two foods of similar nutrient content, only one of which is labeled.

## 8. Strengths and Limitations

We are aware of the limitations and strengths of this narrative review.

Although it sought to include all of the research produced on this topic, some studies may have been overlooked. Another limitation is that during the writing of this review, no scale for its quality assessment was used.

A strength of this paper is that it deepened the research on the FSA-NPS underpinning directive FOPLs, highlighting that a gold standard among NPs is yet to be achieved.

Furthermore, this study sought to provide a wider point of view on the matter, as well as instruments and suggestions for more solid and homogeneous research in the future.

## 9. Discussion

The European Community is looking at the last quarter of 2022 for the harmonization of a single FOPL implementation throughout all EU countries, but if we are willing to make FOPLs a health policy tool, the information given should be easily understandable to the whole population as well as science-based.

Although FOPLs have been studied since 1989, our literature review found research on this topic still in progress. Indeed, regarding the ability of FOPLs to modify purchasing behavior, evidence is limited and suggests a small beneficial effect on “on-site” purchases. Directive FOPLs were found to help consumers rank foods, but they were not associated with a shift in food choices between food categories [29,34,35], while non-directive FOPLs are still under-researched. 

Secondly, in terms of directing individual dietary patterns towards a healthy and sustainable diet, non-directive FOPLs were found to be informative and helpful in increasing consumers’ knowledge [74], while directive ones were strongly capable of helping consumers categorize foods, but the findings on modifications and on a possible impact on dietary patterns appear to be weak [5]. In particular, the correlation between FOPLs and health outcomes, such as a decreased risk of developing NCDs or obesity, was not supported by evidence, since no longitudinal studies were performed. 

Lastly, on reformulating food products by the food industry, research appears to be stalling. On the one hand, many packaged products need to be reformulated [86,87], but while TFPs are impeded by their inherent nature, excluding them from labeling could be confusing for consumers. On the other hand, data on actual reformulation are still pending.

Starting from these findings, we would highlight some limits and gaps found in the current literature:

It is no longer possible to discuss healthy diets without taking the environment and sustainability into consideration.

TFPs, which are often energy-dense foods and representative of local cultures and eating traditions, should be properly studied to avoid a decline in consumption, a subsequent loss in food variability and a breakdown in local economies. There has been a lack of direct study on the primary or secondary prevention effects of any FOPL.

At the same time, we would like to emphasize some other aspects that could be useful for future research:

Directive FOPLs based on FSA-NPS seem to be more immediate and appealing to consumers, but due to the nature of the nutrient profiling algorithm, they do not contextualize the food choices within daily and/or weekly dietary patterns, thus risking promoting an unsustainable or monotonous diet.

Non-directive FOPLs are more informative and useful in enabling consumers to understand the food composition of the products they choose, increasing nutrition knowledge and encouraging a more balanced dietary pattern. On the other hand, they seem to require an interpretative effort that is perceived as time-consuming at the PoP.

Directive and non-directive FOPLs have been developed with different purposes, but they are usually compared to identify the most effective one, so they fail or succeed depending on the primary objective of the study. They should probably be perceived more as two sides of the same coin rather than as competitors.

The use of FOPLs may also reduce interest in mandatory nutrient declarations, which have an important, informative value for the consumer.

Regarding the aim of encouraging members of the food industry to change product formulations, caution must be exercised to ensure that this does not trigger compensatory mechanisms in the industry, leading to the use of “unhealthy” ingredients that are not included in the algorithm, only to achieve a better score that does not reflect the nutritional quality of the food.

In conclusion, it is essential to implement FOPLs with serious, more widespread, structured, programmed policies, coordinated by professionals in the sector who are directly and daily involved with consumer issues, to inform and educate people on how to manage their purchases and on the composition of a healthy and sustainable dietary pattern. It appears clear that there is no one-size-fits-all solution: strategies in food and health policy should be explored within a multi-variable scenario [94]. All scientific gaps should be filled before any decision is made.

## Figures and Tables

**Table 1:** Front-of-pack nutrition labels in the EU and other regions (adapted from Delhomme V., 2021).

Categories of FOP Models			Example of FOP Models		Countries of Use
Nutrient specific labels	Non-directive (Reductive/ Non-interpretative)	Numerical	Reference Intakes	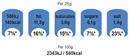	Across Europe
			NutrInform Battery	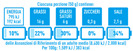	Italy
	Semi-directive (Evaluative/Interpretative)	Color-coded	Multiple Traffic Lights (MTL)	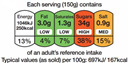	UK, Ireland
		Textual	Warning Labels	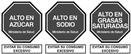	Mexico, Peru, Chile
Summary labels	Directive (Evaluative)	Endorsement Logos	Nordic Keyhole	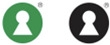	Sweden, Denmark, Lithuania
			Healthy Choices	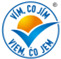	Poland, Czech Republic
		Graded Indicators	Health Star Ratings	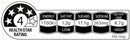	Australia, New Zealand
			Nutri-Score	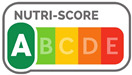	France, Belgium, the Netherlands, Germany, Spain

## Supplementary Materials

The following supporting information can be downloaded at: https://www.mdpi.com/article/10.3390/nu14040771/s1, Table S1: Summary of investigated studies.

## References

[1] Wyrwa J., Barska A. (2017). Packaging as a Source of Information About Food Products. Procedia Eng..

[2] European Parliaments and the Council (2020). Report from the Commission to the European Parliament and the Council Regarding the Use of Additional Forms of Expression and Presentation of the Nutrition Declaration.

[3] Storcksdieck genannt Bonsmann S., Marandola G., Ciriolo E., van Bavel R., Wollgast J. (2020). Front-Of-Pack Nutrition Labelling Schemes: A Comprehensive Review.

[4] Kanter R., Vanderlee L., Vandevijvere S. (2018). Front-of-package nutrition labelling policy: Global progress and future directions. Public Health Nutr..

[5] WHO (2019). Guiding Principles and Framework Manual for Front-of-Pack Labelling for Promoting Healthy Diet. https://apps.who.int/nutrition/publications/policies/guidingprinciples-labelling-promoting-healthydiet/en/index.html.

[6] Ikonen I., Sotgiu F., Aydinli A., Verlegh P.W.J. (2020). Consumer effects of front-of-package nutrition labeling: An interdisciplinary meta-analysis. J. Acad. Mark. Sci..

[7] (2017). WHO. Report of the Commission on Ending Childhood Obesity: Implementation Plan: Executive Summary, Geneva.

[8] (2015). C. Codex Alimentarius Commission. Guidelines on Nutrition Labelling CAC/GL 2-1985, Geneva. https://www.fao.org/fao-who-codexalimentarius/sh-proxy/es/?lnk=1&url=https%253A%252F%252Fworkspace.fao.org%252Fsites%252Fcodex%252FStandards%252FCXG%2B2-1985%252FCXG_002e.pdf.

[9] EFSA (2008). The setting of nutrient profiles for foods bearing nutrition and health claims pursuant to Article 4 of the Regulation (EC) No 1924/2006—Scientific Opinion of the Panel on Dietetic Products, Nutrition and Allergies. EFSA J..

[10] World Health Organization (2020). Manual to Develop and Implement Front-of-Pack Nutrition Labelling: Guidance for Countries on the Selection and Testing of Evidence-Informed Front-of-Pack Nutrition Labelling Systems in the WHO European Region4. https://apps.who.int/iris/bitstream/handle/10665/336988/WHO-EURO-2020-1569-41320-56234-eng.pdf.

[11] Rayner M. (2017). Nutrient profiling for regulatory purposes. Proc. Nutr. Soc..

[12] Hagmann D., Siegrist M. (2020). Nutri-Score, multiple traffic light and incomplete nutrition labelling on food packages: Effects on consumers’ accuracy in identifying healthier snack options. Food Qual. Prefer..

[13] Delhomme V. (2021). Front-of-pack nutrition labelling in the European Union: A behavioural, legal and political analysis. Eur. J. Risk Regul..

[14] World Health Organization/Regional Office for Europe (2013). Vienna Declaration on Nutrition and Noncommunicable Diseases in the Context of Health 2020. https://www.euro.who.int/en/publications/policy-documents/vienna-declaration-on-nutrition-and-noncommunicable-diseases-in-the-context-of-health-2020-2013.

[15] Brinsden H., Lobstein T. (2013). Comparison of nutrient profiling schemes for restricting the marketing of food and drink to children. Pediatr. Obes..

[16] Storcksdieck S., Caldeira S., Wollgast J., Mak T.N. (2017). Comparison of the nutrient profiling schemes of the EU Pledge and the World Health Organization Regional Office for Europe. Eur. Sci. Tech. Res. Rep..

[17] De Edelenyi F.S., Egnell M., Galan P., Druesne-Pecollo N., Hercberg S., Julia C. (2019). Ability of the Nutri-Score front-of-pack nutrition label to discriminate the nutritional quality of foods in the German food market and consistency with nutritional recommendations. Arch. Public Health.

[18] Scarborough P., Rayner M., Stockley L. (2007). Developing nutrient profile models: A systematic approach. Public Health Nutr..

[19] Scarborough P., Boxer A., Rayner M., Stockley L. (2007). Testing nutrient profile models using data from a survey of nutrition professionals. Public Health Nutr..

[20] Arambepola C., Scarborough P., Rayner M. (2008). Validating a nutrient profile model. Public Health Nutr..

[21] (2015). W. R. O. for E. Europe. WHO Regional Office for Europe Nutrient Profile Mode.

[22] Solomon M.R., Hogg M.K., Michael, Askegaard S., Bamossy G. (2006). Consumer Behaviour. A European Perspective.

[23] Samant S.S., Seo H.-S. (2016). Effects of label understanding level on consumers’ visual attention toward sustainability and process-related label claims found on chicken meat products. Food Qual. Prefer..

[24] Taillie L.S., Hall M.G., Popkin B.M., Ng S.W., Murukutla N. (2020). Experimental Studies of Front-of-Package Nutrient Warning Labels on Sugar-Sweetened Beverages and Ultra-Processed Foods: A Scoping Review. Nutrients.

[25] Ronteltap A., Sijtsema S.J., Dagevos H., de Winter M.A. (2012). Construal levels of healthy eating. Exploring consumers’ interpretation of health in the food context. Appetite.

[26] Marinescu V., Fox B., Cristea D., Roventa-Frumusani D., Marinache R., Branea S. (2021). Talking about Sustainability: How the Media Construct the Public’s Understanding of Sustainable Food in Romania. Sustainability.

[27] Persoskie A., Hennessy E., Nelson W.L. (2017). US Consumers’ Understanding of Nutrition Labels in 2013: The Importance of Health Literacy. Prev. Chronic Dis..

[28] Sabbagh C., Boyland E., Hankey C., Parrett A. (2020). Analysing Credibility of UK Social Media Influencers’ Weight-Management Blogs: A Pilot Study. Int. J. Environ. Res. Public Health.

[29] Hall M.G., Lazard A.J., Grummon A.H., Mendel J.R., Taillie L.S. (2020). The impact of front-of-package claims, fruit images, and health warnings on consumers’ perceptions of sugar-sweetened fruit drinks: Three randomized experiments. Prev. Med..

[30] Duffy E.W., Hall M.G., Carpentier F.R.D., Musicus A.A., Meyer M.L., Rimm E., Taillie L.S. (2021). Nutrition Claims on Fruit Drinks Are Inconsistent Indicators of Nutritional Profile: A Content Analysis of Fruit Drinks Purchased by Households With Young Children. J. Acad. Nutr. Diet..

[31] Stoltze F.M., Busey E., Taillie L.S., Carpentier F.R.D. (2021). Impact of warning labels on reducing health halo effects of nutrient content claims on breakfast cereal packages: A mixed-measures experiment. Appetite.

[32] La Berge A.F. (2007). How the Ideology of Low Fat Conquered America. J. Hist. Med. Allied Sci..

[33] Borra S.T., Bouchoux A. (2009). Effects of Science and the Media on Consumer Perceptions about Dietary Sugars. J. Nutr..

[34] Dréano-Trécant L., Egnell M., Hercberg S., Galan P., Soudon J., Fialon M., Touvier M., Kesse-Guyot E., Julia C. (2020). Performance of the Front-of-Pack Nutrition Label Nutri-Score to Discriminate the Nutritional Quality of Foods Products: A Comparative Study across 8 European Countries. Nutrients.

[35] De Santos R.M., Marteache A.H., Toledano L., López A.M., Hernández-Agero T.O., Andicoberry C.A. (2020). Report of the Scientific Committee of the Spanish Agency for Food Safety and Nutrition (AESAN) on infant botulism. Revista Comité Científico AESAN.

[36] Van Tongeren C., Jansen L. (2020). Adjustments Needed for the Use of Nutri-Score in the Netherlands: Lack of Selectivity and Conformity with Dutch Dietary Guidelines in Four Product Groups. Int. J. Nutr. Food Sci..

[37] Shangguan S., Afshin A., Shulkin M., Ma W., Marsden D., Smith J., Saheb-Kashaf M., Shi P., Micha R., Imamura F. (2019). A Meta-Analysis of Food Labeling Effects on Consumer Diet Behaviors and Industry Practices. Am. J. Prev. Med..

[38] Julia C., Blanchet O., Méjean C., Péneau S., Ducrot P., Allès B., Fezeu L.K., Touvier M., Kesse-Guyot E., Singler E. (2016). Impact of the front-of-pack 5-colour nutrition label (5-CNL) on the nutritional quality of purchases: An experimental study. Int. J. Behav. Nutr. Phys. Act..

[39] Fialon M., Egnell M., Talati Z., Galan P., Dréano-Trécant L., Touvier M., Pettigrew S., Hercberg S., Julia C. (2020). Effectiveness of Different Front-of-Pack Nutrition Labels among Italian Consumers: Results from an Online Randomized Controlled Trial. Nutrients.

[40] Cowburn G., Stockley L. (2005). Consumer understanding and use of nutrition labelling: A systematic review. Public Health Nutr..

[41] Drichoutis A., Lazaridis P., Nayga R. (2006). Consumers’ use of nutritional labels: A review of research studies and issues. Acad. Mark. Sci. Rev..

[42] Klopp P., Macdonald M. (1981). Nutrition Labels: An Exploratory Study of Consumer Reasons for Nonuse. J. Consum. Aff..

[43] Deliza R., MacFie H. (1996). The generation of sensory expectation by external cues and its effect on sensory perception and hedonic ratings: A review. J. Sens. Stud..

[44] Tórtora G., Ares G. (2018). Influence of time orientation on food choice: Case study with cookie labels. Food Res. Int..

[45] Just D.R., Payne C.R. (2009). Obesity: Can Behavioral Economics Help?. Ann. Behav. Med..

[46] Hollmann M., Pleger B., Villringer A., Horstmann A. (2013). Brain imaging in the context of food perception and eating. Curr. Opin. Lipidol..

[47] De Araujo I.E., Schatzker M., Small D.M. (2020). Rethinking Food Reward. Annu. Rev. Psychol..

[48] Talati Z., Pettigrew S., Kelly B., Ball K., Dixon H., Shilton T. (2016). Consumers’ responses to front-of-pack labels that vary by interpretive content. Appetite.

[49] Hua S.V., Ickovics J.R. (2016). Vending Machines: A Narrative Review of Factors Influencing Items Purchased. J. Acad. Nutr. Diet..

[50] Siegrist M., Hartmann C., Lazzarini G.A. (2019). Healthy choice label does not substantially improve consumers’ ability to select healthier cereals: Results of an online experiment. Br. J. Nutr..

[51] Van Kleef E., Otten K., Van Trijp H.C.M. (2012). Healthy snacks at the checkout counter: A lab and field study on the impact of shelf arrangement and assortment structure on consumer choices. BMC Public Health.

[52] Schlinkert C., Gillebaart M., Benjamins J., Poelman M.P., De Ridder D. (2020). Snacks and The City: Unexpected Low Sales of an Easy-Access, Tasty, and Healthy Snack at an Urban Snacking Hotspot. Int. J. Environ. Res. Public Health.

[53] Vandevijvere S. (2020). Uptake of Nutri-Score during the first year of implementation in Belgium. Arch. Public Health.

[54] Hamlin R., Hamlin B. (2020). An Experimental Comparison of the Impact of ‘Warning’ and ‘Health Star Rating’ FoP Labels on Adolescents’ Choice of Breakfast Cereals in New Zealand. Nutrients.

[55] Julia C., Méjean C., Touvier M., Péneau S., Lassale C., Ducrot P., Hercberg S., Kesse-Guyot E. (2015). Validation of the FSA nutrient profiling system dietary index in French adults—Findings from SUVIMAX study. Eur. J. Nutr..

[56] Adriouch S., Julia C., Kesse-Guyot E., Méjean C., Ducrot P., Péneau S., Donnenfeld M., Deschasaux M., Menai M., Hercberg S. (2016). Prospective association between a dietary quality index based on a nutrient profiling system and cardiovascular disease risk. Eur. J. Prev. Cardiol..

[57] Adriouch S., Julia C., Kesse-Guyot E., Ducrot P., Péneau S., Méjean C., Assmann K.E., Deschasaux M., Hercberg S., Touvier M. (2017). Association between a dietary quality index based on the food standard agency nutrient profiling system and cardiovascular disease risk among French adults. Int. J. Cardiol..

[58] Deschasaux M., Huybrechts I., Murphy N., Julia C., Hercberg S., Srour B., Kesse-Guyot E., Latino-Martel P., Biessy C., Casagrande C. (2018). Nutritional quality of food as represented by the FSAm-NPS nutrient profiling system underlying the Nutri-Score label and cancer risk in Europe: Results from the EPIC prospective cohort study. PLoS Med..

[59] Donnenfeld M., Julia C., Kesse-Guyot E., Méjean C., Ducrot P., Péneau S., Deschasaux M., Latino-Martel P., Fezeu L., Hercberg S. (2015). Prospective association between cancer risk and an individual dietary index based on the British Food Standards Agency Nutrient Profiling System. Br. J. Nutr..

[60] Julia C., Ducrot P., Lassale C., Fézeu L., Méjean C., Péneau S., Touvier M., Hercberg S., Kesse-Guyot E. (2015). Prospective associations between a dietary index based on the British Food Standard Agency nutrient profiling system and 13-year weight gain in the SU.VI.MAX cohort. Prev. Med..

[61] Sánchez-Villegas A., Martínez-Lapiscina E.H. (2018). A Healthy Diet for Your Heart and Your Brain. The Prevention of Cardiovascular Disease through the Mediterranean Diet.

[62] Takata Y., Shu X.-O., Gao Y.-T., Li H., Zhang X., Gao J., Cai H., Yang G., Xiang Y.-B., Zheng W. (2013). Red Meat and Poultry Intakes and Risk of Total and Cause-Specific Mortality: Results from Cohort Studies of Chinese Adults in Shanghai. PLoS ONE.

[63] Van den Brandt P.A. (2019). Red meat, processed meat, and other dietary protein sources and risk of overall and cause-specific mortality in The Netherlands Cohort Study. Eur. J. Epidemiol..

[64] Sun Q., Pan A., Bernstein A.M., Schulze M.B., Manson J.E., Stampfer M.J., Willett W.C., Hu F.B. (2012). Red Meat Consumption and Mortality: Results from 2 prospective cohort studies. Arch. Intern. Med..

[65] Rohrmann S., Overvad K., Bueno-De-Mesquita H.B., Jakobsen M.U., Egeberg R., Tjønneland A., Nailler L., Boutron-Ruault M.-C., Clavel-Chapelon F., Krogh V. (2013). Meat consumption and mortality—Results from the European Prospective Investigation into Cancer and Nutrition. BMC Med..

[66] Macdiarmid J.I., Whybrow S. (2019). Nutrition from a climate change perspective. Proc. Nutr. Soc..

[67] Poore J., Nemecek T. (2018). Reducing food’s environmental impacts through producers and consumers. Science.

[68] Shukla P.R., Skea J., Buendia E.C., Masson-Delmotte V., Pörtner H.-O., Roberts D.C., Zhai P., Slade R., Connors S., van Diemen R. (2019). IPCC, 2019: Climate Change and Land: An IPCC Special Report on Climate Change, Desertification, Land Degradation, Sustainable Land Management, Food Security, and Greenhouse Gas Fluxes in Terrestrial Ecosystems.

[69] Board S.S., Strazzullo P., Cairella G., Sofi F., Erba D., Campanozzi A., Danesi F., Iacoviello L., Martini D., SINU Scientific Committee (2021). “Front-of-pack” nutrition labeling. Nutr. Metab. Cardiovasc. Dis..

[70] Bonanni A.E., Bonaccio M., Di Castelnuovo A., De Lucia F., Costanzo S., Persichillo M., Zito F., Donati M.B., De Gaetano G., Iacoviello L. (2013). Food Labels Use Is Associated with Higher Adherence to Mediterranean Diet: Results from the Moli-Sani Study. Nutrients.

[71] Navarrete-Muñoz E.M., Torres-Collado L., Valera-Gran D., Gonzalez-Palacios S., Compañ-Gabucio L.M., Hernández-Sánchez S., Garcia-De-La-Hera M. (2018). Nutrition Labelling Use and Higher Adherence to Mediterranean Diet: Results from the DiSA-UMH Study. Nutrients.

[72] Mazzù M.F., Romani S., Gambicorti A. (2021). Effects on consumers’ subjective understanding of a new front-of-pack nutritional label: A study on Italian consumers. Int. J. Food Sci. Nutr..

[73] Mazzù M.F., Romani S., Baccelloni A., Gambicorti A. (2021). A cross-country experimental study on consumers’ subjective understanding and liking on front-of-pack nutrition labels. Int. J. Food Sci. Nutr..

[74] Baccelloni A., Giambarresi A., Mazzù M.F. (2021). Effects on Consumers’ Subjective Understanding and Liking of Front-of-Pack Nutrition Labels: A Study on Slovenian and Dutch Consumers. Foods.

[75] (2021). Information on the Italian Counter Proposal to Nutri-Score: The Nutrinform Battery System. https://nutriscore.blog/2021/03/25/information-on-the-italian-counter-proposal-to-nutri-score-the-nutrinform-battery-system.

[76] EFSA (2021). EFSA Explains Draft Scientific Opinion on a Tollerable Upper Intake Level for Dietary Sugars.

[77] Ducrot P., Méjean C., Julia C., Kesse-Guyot E., Touvier M., Fezeu L.K., Hercberg S., Péneau S. (2015). Objective Understanding of Front-of-Package Nutrition Labels among Nutritionally At-Risk Individuals. Nutrients.

[78] Egnell M., Ducrot P., Touvier M., Allès B., Hercberg S., Kesse-Guyot E., Julia C. (2018). Objective understanding of Nutri-Score Front-Of-Package nutrition label according to individual characteristics of subjects: Comparisons with other format labels. PLoS ONE.

[79] Ducrot P., Méjean C., Julia C., Kesse-Guyot E., Touvier M., Fezeu L., Hercberg S., Péneau S. (2015). Effectiveness of Front-Of-Pack Nutrition Labels in French Adults: Results from the NutriNet-Santé Cohort Study. PLoS ONE.

[80] Carruba M.O., Caretto A., De Lorenzo A., Fatati G., Ghiselli A., Lucchin L., Maffeis C., Malavazos A., Malfi G., Riva E. (2021). Front-of-pack (FOP) labelling systems to improve the quality of nutrition information to prevent obesity: NutrInform Battery vs Nutri-Score. Eat. Weight Disord. Stud. Anorex. Bulim. Obes..

[81] Grunert K.G., Wills J.M., Fernández-Celemín L. (2010). Nutrition knowledge, and use and understanding of nutrition information on food labels among consumers in the UK. Appetite.

[82] Gómez-Donoso C., Martínez-González M., Ángel, Perez-Cornago A., Sayon-Orea C., Martínez M., Bes-Rastrollo J.A. (2021). Association between the nutrient profile system underpinning the Nutri-Score front-of-pack nutrition label and mortality in the SUN project: A prospective cohort study. Clin. Nutr..

[83] Egnell M., Crosetto P., D’Almeida T., Kesse-Guyot E., Touvier M., Ruffieux B., Hercberg S., Muller L., Julia C. (2019). Modelling the impact of different front-of-package nutrition labels on mortality from non-communicable chronic disease. Int. J. Behav. Nutr. Phys. Act..

[84] Carter O.B.J., Mills B.W., Lloyd E., Phan T. (2012). An independent audit of the Australian food industry’s voluntary front-of-pack nutrition labelling scheme for energy-dense nutrition-poor foods. Eur. J. Clin. Nutr..

[85] Van Camp D., Hooker N., Monteiro D.M.D.S. (2010). Adoption of voluntary front of package nutrition schemes in UK food innovations. Br. Food J..

[86] Combet E., Vlassopoulos A., Mölenberg F., Gressier M., Privet L., Wratten C., Sharif S., Vieux F., Lehmann U., Masset G. (2017). Testing the Capacity of a Multi-Nutrient Profiling System to Guide Food and Beverage Reformulation: Results from Five National Food Composition Databases. Nutrients.

[87] Vlassopoulos A., Masset G., Charles V.R., Hoover C., Chesneau-Guillemont C., Leroy F., Lehmann U., Spieldenner J., Tee E.-S., Gibney M. (2017). A nutrient profiling system for the (re)formulation of a global food and beverage portfolio. Eur. J. Nutr..

[88] Galanakis C.M. (2019). Innovations in Traditional Foods.

[89] Askegaard S., Madsen T.K. (1998). The local and the global: Exploring traits of homogeneity and heterogeneity in European food cultures. Int. Bus. Rev..

[90] Becker T.C., Staus A. (2008). European Food Quality Policy: The Importance of Geographical Indications, Organic Certification and Food Quality Insurance Schemes in European Countries. Proceedings of the 2008 International Congress.

[91] Burlingame B., Gitz V., Meybeck A. (2015). Mediterranean Food Consumption Patterns: Diet, Environment, Society, Economy and Health A White Paper Priority 5 of Feeding Knowledge Programme Expo Milan.

[92] Skuras D., Vakrou A. (2002). Consumers’ willingness to pay for origin labelled wine. Br. Food J..

[93] Avermaete T., Viaene J., Morgan E.J., Pitts E., Crawford N., Mahon D. (2004). Determinants of product and process innovation in small food manufacturing firms. Trends Food Sci. Technol..

[94] Marcone M.F., Madan P., Grodzinski B. (2020). An Overview of the Sociological and Environmental Factors Influencing Eating Food Behavior in Canada. Front. Nutr..

