# Supplementary material for: Are Front-of-Pack Labels a Health Policy Tool?
Source: Nutrients. 2022 Feb 11;14(4):771. doi: 10.3390/nu14040771 (PMC8879020; doi:10.3390/nu14040771)
Supplement: Supplementary file 1 [file nutrients-14-00771-s001.zip › nutrients-1572815-supplementary.pdf]

Table S1. Summary of investigated studies

Reference	Study Type	Aim/Purpose	Sample Features	Summary of Results	Limits
Ikonen et al. (2020) [5]	Meta-analysis	To address the existing gaps with a meta-analytic review of all articles examining the effects of health- or nutrition-related FOP labels at the consumer level.	114 Articles	Although FOPLs help consumers to identify healthier products, their ability to nudge consumers toward healthier choices is more limited. Importantly, FOPLs may positively influence not only healthy but also unhealthy products (Halo Effect).	- All the studies included assume consumer awareness of the label<br>- Possibility of failing to identify some publications
Hagmann et al. (2020) [11]		To compare two evaluative FOPLs (MTL and Nutri-Score) in terms of their effect on consumers' healthiness evaluation of salty snacks.	Sample size=1313<br>Sex=53,9%W<br>Age>18	Nutri-Score led to the greatest accuracy in identifying the healthier of two snacks. Both FOPLs were superior to the one with and without the nutrition facts. The perceived usefulness and public support of mandatory implementation were higher for the MTL than for the Nutri-Score label.	- No objective criterion exists for "healthiness" that could be used to compare the effectiveness of the Nutri-Score label and the MTL<br>- It remains an open question whether in real-world shopping situations, healthiness is such an important criterion for consumers when choosing snack foods
Brindsden et al. (2013) [14]		To compare the proposed USA and EU nutrient profiling schemes and three government-led schemes, paying particular attention to the differences in sugar criteria.	Sample size=178 food products	Under the new USA and EU nutrient profiling schemes, 88 (49%) and 73 (41%) of a total of 178 products would be permitted to be advertised, respectively. The US IWG permitted 25 (14%) products; the Ofcom regulations permitted 65 (37%) and the	- The authors came across a number of points of ambiguity categorizing products, and when assessing the products in relation to 'nutrients to promote' criteria<br>- This paper does not compare schemes in respect of the media

Reference	Study Type	Aim/Purpose	Sample Features	Summary of Results	Limits
				Danish Code permitted 13 (7%)	channels, children's age or proportion of viewing audience, or other aspects of the schemes which determine how well the schemes are able to protect children from exposure to the promotion of food and beverages
Szabo de Edelenyi et al. (2019) [16]		To verify the Nutri-Score ability to discriminate the nutritional quality of foods and beverages currently present on the market in Germany and its consistency with German Food-Based Dietary Guidelines (FBDG).	Sample size=8587 foods available on the German market	The classification of foods according to the Nutri-Score was consistent with German FBDG. The NP system underpinning the Nutri-Score was able to display the variability in nutritional quality of foods within the same food groups.	- The main limitation of the study pertains to the use of the Open Food Facts database<br>It wasn't possible to analyze the representativeness of the sample of foods retrieved, either in terms of number of products or market share
Scarborough et al. (2007) [17]		To describe a systematic and logical approach to nutrient profiling.		The NP model developed for this paper calculates scores for foods using a simple equation. It enables breakfast cereals to be compared with each other and with other foods eaten at breakfast.	- The model considers only a limited number of nutrients, and does not provide a complete picture of which foods are most likely to contribute towards a healthier breakfast
Scarborough et al. (2007) [18]	Survey	To compare nutrient profile models with a standard ranking of 120 foods from the results of a survey of the views of nutrition professionals.	Sample size=700 nutrition professionals	Models SSCg3d and WXYfm rank and categorise foods in accordance with the views of nutrition professionals.	- - Using the standard ranking of foods derived from a survey of nutrition professionals to assess the validity of different NP models should therefore be used with caution
Arambepola et al. (2008) [19]		To assess the validity of nutrient profiling Model	Sample size=1117 18<Age<65	Model WXYfm showed good construct validity: the energy	- Absence of a gold standard for

Reference	Study Type	Aim/Purpose	Sample Features	Summary of Results	Limits
		WXYfm – developed for the purpose of regulating the promotion of ‘less healthy’ foods to children.		intake from ‘less healthy’ foods amongst subjects with the least healthy diets was nearly twice the energy intake from ‘less healthy’ foods amongst the subjects with the healthiest diets.	testing the criterion validity of NP models<br>- The healthiness of a diet is clearly not just a function of the ‘healthiness ’of the foods of which it is composed
Samant et al. (2016) [22]		To determine the effect of label education on consumers’ purchase behavior, measured with respect to their visual attention to label claims on chicken products.	Sample size=58	Participants in the Control group (CNTL) examined the appearance of the meat product more often and longer than those in the Experimental group (HLU) did. In contrast, participants in the HLU group looked at the label claims associated with sustainability and process more often and longer than did those in the CNTL group.	- The number of participants was not large enough for providing conclusive evidence
Dréano-Trécant et al. (2020) [24]		To evaluate the applicability of the Nutri-Score in various EU countries, regarding its ability to discriminate the nutritional quality of foods and its consistency with national dietary recommendations.	Sample size = 11347 foods and beverages	The Nutri-Score demonstrated high discriminating ability for all food groups, with similar trends in the eight countries, and consistency with nutritional recommendations.	- Data from EUROFIR were retrieved from different sources<br>- The somewhat arbitrary aspect of the measurements of the discriminating performance of the FoPL and the consistency with dietary guidelines in the absence of a consensual indicator to which to compare the performance of nutrient profiling
van Tongeren et al.		To investigate the	Sample size=2299	For the product groups	

Reference	Study Type	Aim/Purpose	Sample Features	Summary of Results	Limits
(2020) [26]		applicability of Nutri-Score in the Netherlands, by comparing Nutri-Score with Dutch Choices criteria and Dutch dietary guidelines in different product groups.	food products	*Cheese, Ready meals, Soups and Meal sauces*, there is an uneven distribution of the products among different Nutri-Scores. The Nutri-Scores of a great amount of products are not in line with Dutch Choices criteria and dietary guidelines, and there is no high correlation between Nutri-Score and health influencing nutrients in most product groups.	
Shangguan et al. (2018) [27]	Meta-analysis	To assess the independent effect of labeling without other major legislative or regulatory interventions, as independently adjudicated by two investigators.	Sample size=60 articles	Food labeling reduces consumer dietary intake of selected nutrients and influences industry practices to reduce product contents of sodium and artificial trans fat.	- Many of the nonrandomized interventions were natural experiments, increasing generalizability<br>- Studies not included here assessed front-of-pack grading systems, such as Health Star Ratings and Nutri-Score, findings of these studies have been inconsistent and often evaluated knowledge or attitudes rather than actual purchasing or consumption
Julia et al. (2016) [28]		To investigate the impact of the 5-CNL on the nutritional quality of purchases in experimental supermarkets.	Sample size=300<br><br>Sex=73M;227F<br><br>Age>18	The 5-CNL FOP nutrition label may have a limited impact on purchases, leading to healthier food choices in some food categories such as sweet biscuits.	- The impact of the label was differential across categories of foods<br>- The absence of usual disturbances present in shops (e.g. the absence of other

Reference	Study Type	Aim/Purpose	Sample Features	Summary of Results	Limits
					shoppers or promotions) may have influenced the behavior of the participants
Fialon et al. (2020) [29]		To investigate Italian consumers' objective understanding of the Nutri-Score and four other FoPLs (Health Star Rating system (HSR), MTL, RIs, warning symbol), as well as the effect of these FoPLs on food choices.	Sample size=1032<br><br>Sex=50%F<br><br>Age>18	The Nutri-Score demonstrated the highest overall performance in helping consumers to correctly rank the products according to their nutritional quality compared to the reference intakes.	- Italian participants were recruited online using quotas, which would limit the generalizability of the findings to the population as a whole<br>- Participants did not have access to the nutritional composition of the products used in the study<br>- The study was conducted as an online experiment and not in a real shopping situation
Hamlin et al. (2020) [35]		To investigate the performance of the red, octagonal Vienna Convention traffic 'STOP' sign as a front of pack (FoP) warning nutritional label compared with that of a single star (low nutritional value) Australasian Health Star Rating (HSR).	Sample size=1032<br><br>Sex=39%M;58%F ;3%GF<br><br>16<Age<18	VCSTOP label significantly influenced purchase intention by suppressing purchase intention when it was present and the VCSTOP outperformed the HSR label.	- The primary limitation of this research relates to the presence of interaction in the results. The presence of interaction may be due to a field methodological feature of this school-based research, or it may be more fundamental to the labels, products and diads themselves.
Mazzù et al. (2020) [53]		To assess the clear comprehension by consumers of the information provided by the NutrInform Battery FOPL scheme.	Sample size=200<br><br>Sex=68%F<br><br>Age>18	Consumers found the NutrInform Battery more informative and helpful than the Nutri-Score in terms of their understanding of the product composition.	- Various phases characterize the consumer's decision-making process. In this study, each of them is presented, but the focus was only on subjective understanding and liking

Reference	Study Type	Aim/Purpose	Sample Features	Summary of Results	Limits
Mazzù et al. (2021) [54]		To test the effect that the new enriched informative label NutrInform Battery and the summary label Nutri-Score have on subjective comprehension and liking across seven European countries (France, Germany, Greece, Italy, Portugal, Romania and Spain).	Sample size=2776<br><br>Age>18	NutrInform Battery obtained the highest performance across countries and showed limited impact on socio-cultural differences.	- Participants to have access to back-of-pack information regarding the nutritional composition of the products used in the study<br>- Subjective understanding and liking are just two dimensions that influence consumers ' reactions to FOPL
Gomez-Donoso et al. (2020) [61]	Prospective cohort study	To prospectively assess the association between the FSAm-NPS and mortality rate in a Spanish cohort of university graduates.	Sample size=20503<br><br>Mean Age=38ys	The consumption of food products with a higher FSAm-NPS score (lower nutritional quality) was associated with a higher rate of all-cause and cancer mortality in a large prospective cohort of Spanish, middle-aged university graduates.	- The FFQ was not specifically designed to collect data about pre-packaged food products that qualify for a Nutri-Score label, and it only covers a small sample of the food products available for consumption<br>- Graduates collaborating with a study focused on lifestyle and health, tend to be more health-conscious compared to the general population
Egnell et al. (2019) [62]	Experimental study + Observational study	To investigate the effects of five different FoPLs (Nutri-Score, Health Star Rating system, Multiple Traffic lights, Reference intakes and SENS (Système d'Etiquetage Nutritionnel Simplifié)) on the nutritional quality of household purchases.	Sample size=691 (experimental brench) + 81421 (observational brench)	Approximately 3.4% of all deaths from diet-related NCDs was estimated to be avoidable if the Nutri-Score was used by participant during the study . The remaining FoPLs likewise resulted in mortality reduction, although to a lesser extent: Health Star Rating system (2.8%), Reference	- Purchases were performed at the household level, and it wasn't possible to link the purchased products specifically to the consumer

Reference	Study Type	Aim/Purpose	Sample Features	Summary of Results	Limits
				Intakes (1.9%), Multiple Traffic Lights (1.6%), and SENS (1.1%).	
Skuras et al. (2002) [68]		To identify which consumer's characteristics and other variables related to the characteristics of the region have an influence on the values derived for willingness to pay.	Sample size= 744<br><br>Age>18	Non-quality wine consumers are willing to pay double the price of a bottle of normal table wine if the alternative provides for a guarantee of the place of origin of the wine.	- The place of origin marketing cue is not a straightforward quality attribute<br>- Consumers may not be totally aware of what ``certification of place of origin "implies for the product's overall quality characteristics
Carter et al. (2012) [70]		To assess the Australian food industry self-regulated front-of-pack (FOP) food labelling system -the Daily Intake Guide (DIG).	Sample size=728 EDNP	The DIG was displayed on 66% of audited EDNP products but most of these (75%) did not report SFA and sugar content. Branded products not displaying fat and sugar content contained on average 10-times more saturated fat than those displaying such and nearly twice as much sugar.	
